# Reevaluating fibromyalgia diagnosis: a proposal to integrate deep tendon reflex responses into current criteria

**DOI:** 10.1007/s00296-025-05846-y

**Published:** 2025-04-02

**Authors:** Ilke Coskun Benlidayi, Ceren Ornek, Volkan Deniz, Aylin Sariyildiz

**Affiliations:** 1 Department of Physical Medicine and Rehabilitation, Faculty of Medicine, Cukurova University, Adana, Turkey; 2 Department of Physical Medicine and Rehabilitation, Muş Government Hospital, Muş, Turkey; 3 Department of Physiotherapy and Rehabilitation, Tarsus University Faculty of Health Sciences, Mersin, Turkey

**Keywords:** Fibromyalgia, Hyperreflexia, Reflex, knee, abnormal, Reflex, triceps, abnormal, Diagnosis

## Abstract

Fibromyalgia is a complex condition characterized by widespread pain, fatigue, and various other symptoms. The symptoms of fibromyalgia overlap with numerous other disorders (e.g., infections, chronic fatigue syndrome), which makes diagnosis challenging. Existing diagnostic criteria for fibromyalgia rely particularly on subjective patient reports. Such a limitation may lead to both missed diagnoses and potential overdiagnoses. Recent research has identified significantly increased deep tendon reflex (DTR) responses in patients with fibromyalgia. The results also demonstrated the potential for DTR examination to help with the diagnostic process, particularly with ruling out fibromyalgia. The potential underlying mechanisms behind the increased DTR responses in fibromyalgia are central nervous system dysregulation, altered muscular properties, autonomic nervous system dysfunction, and accompanying conditions such as hypomagnesemia and anxiety. By integrating DTR responses into current diagnostic criteria sets, physicians may more effectively differentiate fibromyalgia from other conditions and avoid the pitfalls of misdiagnosis, as well as overdiagnosis. The use of DTR testing in the diagnostic evaluation of fibromyalgia shows promise. Yet, it has both advantages and limitations. The potential benefits of this approach include improved diagnostic accuracy, but challenges remain in its low specificity. This means that hyperreflexia testing alone is not definitive in diagnosing fibromyalgia. Nonetheless, given the high sensitivity, a decreased DTR response could still contribute to ruling out fibromyalgia.

## Introduction

Is fibromyalgia very easy to diagnose? If so, does this “simplicity” make fibromyalgia prone to overdiagnosis? If not, what are the challenges involved in reaching an accurate diagnosis? These questions, along with the findings of our recent research, which demonstrated increased deep tendon reflex (DTR) responses in patients with fibromyalgia [1], have paved the way for us to discuss the current diagnostic methods for fibromyalgia and propose integrating a clinical finding into the existing diagnostic criteria.

There are complexities involved in diagnosing fibromyalgia, which is a condition with a broad and overlapping symptom profile. Several diagnostic criteria sets have been proposed by researchers so far [2–6]. The diagnosis is often based on subjective reports of widespread pain, fatigue, sleep and mood disturbances, which can easily be mistaken for other conditions (e.g., chronic viral hepatitis, hypothyroidism, chronic fatigue syndrome, major depressive disorder). Although several tools are regarded as a convenient method for documenting and assessing disease-related symptoms [7], the lack of a clear clinical diagnostic test or a laboratory analysis (i.e., a biomarker) for fibromyalgia increases the likelihood of both missed diagnoses and potential overdiagnoses.

We recently published a case-control study on the use of DTR in fibromyalgia diagnosis [1]. The clinical and electrophysiological study examined one muscle from the upper extremity (triceps brachii) and one from the lower extremity (rectus femoris), and assessed their DTR responses. The comparison of electromyographic, kinematic, and electromechanical DTR responses between the patient and control groups demonstrated significantly higher amplitude, higher sagittal acceleration and angular velocity in the patient group. Moreover, the electromechanical delay was significantly shorter in patients with fibromyalgia. Our study revealed that patients with fibromyalgia exhibit increased clinical and electromyographic DTR responses. As another important finding, normoactive/hypoactive DTR responses appeared to rule out fibromyalgia with 94.1% sensitivity and 61.3% specificity. Thus, a conclusion was made that normal or decreased DTR response may probably serve as a rule-out criterion for fibromyalgia [1]. This current review aims to thoroughly explore the background and pathophysiological processes that may explain the potential utility of this technique in fibromyalgia diagnosis.

The above-mentioned results position DTR examination as a clinical sign that could probably be used to diagnose, and particularly to exclude (given the higher sensitivity), fibromyalgia. Such an addition to the existing literature becomes even more crucial given the challenges that healthcare professionals experience in the diagnosis of fibromyalgia. A clinical examination could help with distinguishing fibromyalgia from other disorders as well as avoiding overdiagnosis.

This review aimed to discuss (i) the potential explanations for increased DTR responses in patients with fibromyalgia, (ii) the limitations of existing diagnostic criteria, and (iii) the feasibility of incorporating DTR examination into current diagnostic criteria.

## Search strategy

The review applied a search strategy proposed for writing narrative reviews [8]. According to the recommendations, the search included multiple databases/search engines (PubMed/MEDLINE, Web of Science, Scopus, and DOAJ). The keywords used in the search included (i) fibromyalgia, (ii) hyperreflexia, (iii) reflex, (iv) abnormal deep tendon reflex, and (v) abnormal reflex, as well as their combinations. Observational studies and randomized controlled trials published within the last 10 years and written in English language were given priority. Congress abstracts and theses were excluded. Additionally, included articles’ reference lists were searched through to identify further relevant papers.

## Challenges associated with the current methods/criteria used for diagnosing fibromyalgia

Various classification criteria have been developed to assist in diagnosis and establish a common terminology [9]. The first classification criteria for fibromyalgia were published in 1990 by the American College of Rheumatology (ACR). These criteria required the presence of chronic widespread pain lasting more than three months, along with tenderness in at least 11 of 18 specific tender points [2]. Nevertheless, the tender point examination had limitations, such as being difficult to apply in clinical practice and being open to interpretation. Additionally, a reduction in the number of tender points due to any reason (e.g., recovery) and the lower prevalence of tender points in men might lead to failure in meeting the classification criteria [3, 10]. On the other hand, the 1990 criteria did not include important components of fibromyalgia, such as fatigue, sleep problems, cognitive issues, and somatic symptoms. Thus, the ACR revised the criteria in 2010. The tender point examination was removed [3, 11]. The number of painful body regions and the severity of fatigue, unrefreshing sleep, cognitive problems, and somatic symptoms in the past week were considered. Patients whose symptoms had persisted for more than three months and who had a Widespread Pain Index score of ≥ 7 and a Symptom Severity Scale score of ≥ 5, or a Widespread Pain Index score of 3–6 and a Symptom Severity Scale score of ≥ 9, with no other conditions explaining the pain, were classified as having fibromyalgia [3]. In 2011, to increase the applicability in epidemiological studies, clinician-assessed somatic symptoms were removed and self-reported symptoms (headaches, abdominal pain, and depression) over the past six months were added [4]. In 2013, Bennett et al. proposed alternative criteria that included additional features such as hand and foot pain, stiffness, tenderness to touch, and sensitivity to environmental stimuli [12]. However, compared to the 2010 criteria, these did not provide an additional benefit in distinguishing fibromyalgia from other chronic widespread pain conditions [13]. The 2010 criteria were more sensitive than the 1990 criteria but had a higher false-positive rate and led to misclassification of regional pain syndromes. In 2016, a generalized pain criterion (pain in at least four of five defined body regions) was added to address this issue. Additionally, the Widespread Pain Index threshold was raised from 3 to 4, and it was emphasized that fibromyalgia is not a diagnosis of exclusion [5]. Ahmed et al. evaluated the performance of 2016 ACR criteria and concluded that patients diagnosed by only the ACR 2016 criteria (and not by the ACR 1990) had high probability of having another concomitant comorbidity [14]. In 2018, Arnold et al. proposed that fibromyalgia could be diagnosed if there was pain in at least six of nine body regions, along with moderate or greater severity of fatigue or sleep disturbances persisting for more than three months. They also emphasized that fibromyalgia is not a diagnosis of exclusion but highlighted the importance of clinical evaluation to rule out other conditions that could explain the symptoms [15].

Currently, the reliance of fibromyalgia classification criteria on patient self-report and the lack of a standardized, objective, and practical physical examination finding that could assist in diagnosis lead to challenges in diagnosis [16]. Furthermore, the fact that fibromyalgia is not a diagnosis of exclusion but also lacks specific classification criteria may lead to overlooking other comorbid conditions that could explain the symptoms [17]. This may also result in overdiagnosis/false positivity issues. Another limitation is that the Symptom Severity Scale evaluates only certain symptoms of fibromyalgia and does not fully reflect the complexity of the disease [18]. On the other hand, increasing the complexity of the criteria may reduce their applicability in clinical practice [17].

## The components and pathway of deep tendon reflex response

The DTR is a monosynaptic reflex arc. This reflex arc is controlled by higher centers, the corticospinal and other descending pathways. Its components include the (i) sensory receptor, (ii) afferent neuron, (iii) integration center, (iv) efferent neuron, and (v) effector organ. 

### Sensory receptor

When a muscle tendon is tapped with a reflex hammer, the muscle spindle detects sudden changes in muscle length and stretch [19]. Muscle spindles are sensory proprioceptors of the muscle that are sensitive to the length of the muscle. The center is the receptor part of the muscle spindle and is not contractile, while the ends (the polar region) are contractile [20]. The intrafusal fibers are surrounded by sensory nerve endings that become 1a afferents to the dorsal root ganglion [21]. When the muscle is stretched (lengthened), the stretch is perceived by the receptors in this region and activates the sensory receptors [20]. Muscle spindles send signals to the spinal cord through the dorsal horn. These signals follow four different paths: (i) to the brain (cortex) which allows for the awareness of the muscle stretch, (ii) directly to an alpha motoneuron, which causes an immediate contraction of the same muscle (agonist) to neutralize the stretch, (iii) to an inhibitory interneuron, which would reduce the antagonist muscle’s activity and thereby allow the agonist to contract more easily, and (iv) to the cerebellum (via the dorsal spinocerebellar tracts), which helps with balance and coordination [22].

### Afferent neuron

Group Ia sensory fibers, which transmit the stretch signal to the spinal cord, are the primary afferent neurons involved in the DTR. The bodies of afferent neurons are located in the dorsal root ganglion [23]. The peripheral axon extends to the muscle spindle and the central axon projects into the spinal cord’s ventral horn, where it synapses directly with alpha motor neurons. The amplitude of DTR is related to the excitability of motor neurons and the sensitivity of muscle spindles. Our recent research has confirmed that patients with fibromyalgia exhibit significantly higher reflex amplitudes [1]. The increased facilitation of the DTR reflects enhanced gamma activity, which regulates the sensitivity of muscle spindles. With enhanced activation of gamma motor neurons, intrafusal muscle fibers contract, so that only a small stretch is required to activate spindle sensory neurons and the stretch reflex [24]. This is mediated by acetylcholine [19].

### Integration center

Within the anterior horn of the spinal cord (lamina IX), the afferent neuron connects directly with the efferent neuron (alpha motor neuron) [23, 25]. In the monosynaptic reflex arc, the afferent neuron monosynaptically stimulates the alpha motor neurons of the agonist muscle. The activating neurotransmitter at the spinal level is glutamate [19].

### Efferent neuron

The efferent neuron (alpha motor neuron) travels through the ventral root and carries the response signal back to the muscle and the effector skeletal muscle contracts (shortens in length) in response to the signal, which constitutes the reflex [19].

### Effector organ

When muscle contraction occurs, the tension in the muscle spindle decreases, the frequency of action potential generation decreases, and the reflex diminishes [19].

The reduction or absence of DTR responses indicates a lesion within the reflex arc. Peripheral neuropathy caused by diabetes, alcoholism, uremia, amyloidosis, or toxins, as well as conditions like hypothyroidism, hypothermia, peripheral nerve injuries, and other diseases or conditions affecting the lower motor neuron, result in decreased or absent DTR responses [19].

Increased DTR responses, on the other hand, refer to a lesion/injury above the level of the spinal reflex pathways-in the upper motor neuron (brain, brainstem, or spinal cord). Consequently, the descending motor pathways that inhibit the reflex arc are thought to be disrupted [26]. The mechanism relies on gamma motor neurons. The intrafusal fibers (the contractile element) of the muscle spindle adjust their sensitivity based on the input they receive from gamma motor neurons, which are located in the anterior horn and controlled by the central nervous system (cortex, cerebellum, and brainstem). The motor innervation (provided by gamma motor neurons) of a sensory structure (muscle spindles), thereby enables these supraspinal structures to set and regulate the sensitivity of the spindle. The higher centers (the cortex in particular) get sensory information from the muscle spindles and, in turn, control the amount and quality of information through the gamma motor neurons [22]. This is particularly important for a better understanding of the supraspinal centers’ role in hyperreflexia (discussed below).

## Potential mechanisms underlying altered deep tendon reflex responses in fibromyalgia

### Central nervous system dysregulation

Muscle spindle apparatus is the major sensory organ embedded in the skeletal muscles and the main determinant of DTR. The gamma motor neuron, an efferent of the muscle spindle, innervates the polar regions of the muscle spindles [20]. The neurotransmitter active in this region is acetylcholine. The body of the gamma motor neurons is located in the anterior horn of the spinal cord and is activated by several central nervous system regions including the cortex, cerebellum, and various brainstem nuclei [21]. These supraspinal centers also receive sensory information from the muscle spindles and control the amount and quality of information received through the gamma motor neuron. In other words, the sensitivity and responsiveness of the muscle spindle are regulated by the gamma motor neurons and the supraspinal centers that stimulate them [23]. In terms of cortical centers, the majority of gamma motor neuron activity is controlled by Primary Motor Cortex (M1), Premotor Cortex, and Supplementary Motor Area. M1 sends descending commands via the corticospinal tract, influencing gamma motor neurons indirectly. Premotor Cortex and Supplementary Motor Area modulate movement planning and postural adjustments, affecting gamma motor neuron activity. Any central dysregulation may affect DTR response. Central nervous system dysregulation has been determined as one of the main drivers of fibromyalgia pathogenesis [27]. In this regard, increased DTR response in fibromyalgia could be attributed to hyperexcitability of spinal reflex arcs/central sensitization and disrupted inhibitory control.

Neuroimaging studies have shown alterations in cerebral microstructural integrity in patients with fibromyalgia [28]. Current evidence indicates that patients with fibromyalgia exhibit abnormal cerebral activation during external stimuli [29]. This may be due to the anomalies in gray matter morphology, regional cerebral blood flow, cortical thickness, functional connectivity at rest, and neurotransmitter concentration [30–33] in patients with fibromyalgia. Potential metabolic, neurochemical, and morphological changes have been studied in relation to brain areas related to sensory processing and pain in fibromyalgia [34–36]. On the other hand, such alterations may involve components of the corticospinal tract, which plays a role in regulating DTR responses. For example, the primary motor cortex (M1), which has been shown to play a role in the maintenance of chronic pain [37], is also one of the main areas from which the corticospinal tract originates. Given the importance of corticospinal tract in DTR responses, altered baseline M1 activity could contribute to the increased DTR responses observed in patients with fibromyalgia. The central nervous system can influence the DTR response through the gamma motor neurons, which are responsible for the sensitivity of DTR.

The reticulospinal tract, an important descending motor pathway, controls tonus and excessive reflex activities by exciting inhibitory interneurons in the spinal cord. The dysfunction of this tract leads to the loss of descending inhibitory input [38]. Reticulospinal tract originates from the brainstem. A potential dysfunction of this area could be one of the underlying factors of hyperreflexia in fibromyalgia. A study by Ioachim et al. demonstrated significant differences in brainstem/spinal cord network connectivity between patients with fibromyalgia and healthy individuals [39]. Voxel-based morphometry analysis performed by Fallon et al. revealed that patients with fibromyalgia had decreased local grey matter volumes and associated geometric shape alterations in the brainstem (pons) and left precuneus [40]. Alterations in spinal and/or brainstem interneuronal circuits may contribute to the dysfunction of descending pain control mechanisms in fibromyalgia [41, 42].

#### Hyperexcitability of spinal reflex arcs/central sensitization

One of the hypotheses thought to play a role in the pathogenesis of fibromyalgia is central sensitization. This refers to the increased excitability of the neurons in the dorsal horn of the spinal cord, which, in turn, heightened spontaneous neuronal activity, expanded receptive fields, and increased responses to the impulses transmitted by both small- and large-fiber sensory afferents [43]. Increased central nervous system sensitivity could alter the regulation of DTR responses in fibromyalgia. It is well-known that the monosynaptic DTR arc is influenced by the balance between excitatory and inhibitory neurons in the spinal cord. Although central sensitization primarily occurs in the dorsal horn, glial activation and inflammatory mediators may affect both dorsal and ventral horn neurons in chronic pain conditions, and the cross-talk between sensory and motor neurons. Thus, an enhanced DTR response may be considered as a potentially expected finding in patients with fibromyalgia. In this context, Tahabit et al. investigated spinal excitability in patients with fibromyalgia using the Hoffman’s reflex [43]. They observed that maximum baseline-to-peak amplitudes of H and M waves (Hmax/Mmax) increased significantly in patients with fibromyalgia compared to controls. Hmax/Mmax ratio is often used as a marker of the proportion of motor neurons activated by the H-reflex in the motor neuronal pool, which consists of all the alpha motor neurons that innervate a specific muscle and is controlled by various supraspinal centers. The results supported the hypothesis that the neurons in the spinal cord are hyperexcitable in fibromyalgia [43].

Fibromyalgia is associated with deficits in intracortical modulation involving both gamma aminobutyric acid (GABA)ergic and glutamatergic mechanisms [44]. Imbalance in excitatory and inhibitory neurotransmitters also at the spinal level can contribute to altered reflex modulation. Central sensitization could be the factor underlying increased DTR responses in fibromyalgia. Inflammation is a key factor in nerve sensitization and the role of inflammation in the pathogenesis of fibromyalgia has attracted considerable attention during the last decade [45]. Research has indicated elevated levels of IL-6, IL-1β, IL-8, and/or tumor necrosis factor (TNF)-α in fibromyalgia [46–49]. Increased expression of proinflammatory cytokines may alter reflex responses. It has been demonstrated that pro-inflammatory cytokine IL-1β plays a role in amplifying the pressor response to muscle contraction and tendon stretch in rats [50].

#### Disrupted inhibitory control

The brain regulates the sensitivity of the stretch reflex to ensure normal muscle tone. Dysregulation of descending inhibitory pathways from the brain could lead to less modulation of reflex arcs, which amplifies DTR responses [51]. Glass et al. found that patients with fibromyalgia showed less brain activation in cortical structures in the inhibition network [52]. Similarly, Jensen et al. showed that patients with fibromyalgia had less functional connectivity in the pain inhibitory network of the brain [53]. Descending reticulospinal projections from the brainstem’s reticular formation inhibit spinal motor neurons, modulating the stretch reflex to optimize muscle tone. Following an upper motor neuron lesion, this inhibitory influence is typically lost through an unclear mechanism, leading to stretch reflex hyperactivity [51].

Damage to an inhibitory cortical structure such as the pyramidal tract may be responsible for increased DTR in fibromyalgia. A study by Salerno et al. evaluated the motor cortex by single and double magnetic stimulation in patients with fibromyalgia [54]. Although the pyramidal tract itself was not impaired, adjacent cortical cells were found to be involved in fibromyalgia [54]. The corticospinal tract includes more than a million myelinated axons on each side. Two thirds of these axons arise from the premotor areas of the frontal lobe and the primary motor cortex. The remaining one third arise from the primary somatosensory cortex (parietal lobe) and synapse in the dorsal horn of the spinal cord. The lateral corticospinal tract is primarily involved in the regulation of DTRs by exerting strong inhibitory control over spinal motor neurons [51].

Cardinal et al. hypothesized that a deteriorated function of cortical inhibition, the dysfunction of the inhibitory descending pain modulatory systems and serum brain-derived neurotrophic factor could differentiate fibromyalgia from major depressive disorder [55]. The inhibitory potency of the descending pain modulatory systems evaluated by the change on the Numerical Pain Scale during conditioned pain modulation test was lower in patients with fibromyalgia compared to those with major depressive disorder [55]. A lower function of the descending pain modulatory systems, along with a higher level of brain-derived neurotrophic factor was shown to differentiate from depression. However, both of these potential markers cannot easily measured/achieved in clinical practice. Instead, increased DTR responses could be used easily as a potentially clinical marker for the deteriorated function of cortical inhibition and the differential diagnosis of fibromyalgia [55].

### Autonomic nervous system dysfunction

Imbalances in the autonomic nervous system are commonly reported in fibromyalgia [56]. Such an imbalance may affect DTR modulation.

#### Alterations in sympathetic activity

The sympathoneural system includes nerve networks, which begin in the locus coeruleus and other cell groups in the medulla and pons, heart, blood vessels, reticuloendothelial organs, and salivary/sweat glands. The main neurotransmitter functioning in this system is norepinephrine. The sympathetic response’s adrenomedullary component includes the cells in the adrenal medulla that release epinephrine, in particular, in response to preganglionic spinal input. Several studies have assessed various aspects of the sympathetic nervous system function in fibromyalgia. These aspects included muscle sympathetic activity, microcirculation, heart rate variability, and endogenous pain modulation [57]. Fibromyalgia patients revealed significantly higher sympathetic skin response amplitude values and lower latency in electroneuromyography evaluation [58]. In general, sympathetic system activity has been associated with an increased release of IL-8 concentrations, which were found to be higher in the cerebrospinal fluid of patients with fibromyalgia. Overall, although there are some conflicting results, patients with fibromyalgia seem to have increased sympathetic activity compared to healthy controls [59] and fibromyalgia is associated with sympathetically dominant dysautonomia [60]. It was demonstrated that the higher the sympathetic drive to the heart/vessels, the higher the magnitude of chronic pain in fibromyalgia [61]. This hyperactivity could also influence neuromuscular excitability and intensify DTR responses.

Another hypothetical explanation involves dorsal root ganglions. The neurons in the dorsal root ganglion, which are close to sympathetic axons, could likely be spontaneously active. Sympathetic fibers could increase excitability and induce spontaneous activity in sensory neurons by releasing sympathetic neurotransmitters. To form a vicious cycle, spontaneously active dorsal root ganglion neurons may attract sympathetic fibers, possibly through the release of neurotrophic factors [62]. In fibromyalgia, chronic pain input to the dorsal root ganglion, along with peripheral sensitization, could also trigger spontaneous activity in the dorsal root ganglia and their attraction to sympathetic fibers.

#### Reduced parasympathetic modulation

Imbalances in parasympathetic activity could fail to balance the effects of the sympathetic system. Research has indicated that there is a disharmony between sympathetic and parasympathetic systems in patients with fibromyalgia [63]. Such a condition may potentially result in altered reflex responses in fibromyalgia.

### Altered muscular properties

#### Increased baseline muscle activity

Chronic muscle stiffness or altered tone may impact DTR responses. Golgi tendon organs, which lie in the muscle tendon and are coupled in series with the extrafusal muscle fibers, convey information about muscle tension [21]. In patients with fibromyalgia, increased baseline muscle activity may lead to heightened muscle tone and, consequently, greater tendon tension, potentially exaggerating reflex contractions when the muscle tendon is tapped. An early study investigating muscle activity in patients with fibromyalgia did not provide evidence of increased baseline muscle activity [64]. However, more recent studies have reported findings suggesting the opposite [65, 66]. Klaver-Krol et al. demonstrated that, in patients with fibromyalgia, electromyographic activity and muscle membrane propagation speed increased at baseline and during various joint movements [65]. This heightened muscle membrane activity suggests dysregulation originating from higher neural centers [65]. In addition, another study revealed that fibromyalgia patients exhibited elevated muscle activity in situations involving imposed stress, including sympathetic activation and putative anticipatory stress [66]. Chronic stress can increase the central sympathetic outflow and alter the immune response [67]. These findings collectively indicate that increased baseline muscle activity may be further amplified under imposed stress conditions, including sympathetic activation and putative anticipatory stress in fibromyalgia.

Peripheral sensitization in fibromyalgia may further contribute to an increased muscle response. Peripheral sensitization is characterized by reduced pain thresholds and hypersensitivity of local nociceptors. Moreover, hypersensitivity to sensory stimuli is not limited to nociceptive input, but includes other sensory domains including sound, sense of smell, and light [68, 69]. Thus, a similar phenomenon may also apply to the reflex response threshold and sensitivity of sensory receptors of the muscles (muscle spindles). Thabit et al. suggested that the potential reduction in the nociceptive thresholds of neurons in the dorsal horn might be induced by peripheral sensitization [43]. Overall, fibromyalgia could be regarded as a condition driven by sensitization of the central nervous system induced via peripheral sensitization [43].

#### Altered soft tissue biomechanical properties

Altered soft tissue biomechanical properties such as muscle stiffness and elasticity may mechanically amplify DTR responses. Increased myofascial stiffness may lead to reduced muscle stretch tolerance and decreased tendon tension, potentially resulting in an enhanced DTR response by altering muscle spindle sensitivity [70]. Numerous studies have provided evidence that muscle stiffness is increased in fibromyalgia patients, as assessed both through self-reported measures [71] and advanced objective methodologies [72, 73]. In fibromyalgia, decreased physical activity levels, increased anxiety, heightened sympathetic activation, and elevated baseline muscle activity are thought to contribute to altered muscle stiffness.

### Accompanying conditions

#### Anxiety

Anxiety has been shown to amplify muscle spindle sensitivity [74]. One possible explanation for this heightened sensitivity is that the spindle provides stronger and more detailed feedback to the brain in response to anxiety. The other could be a heightened state of neural activity associated with anxiety [75]. Additionally, chronic stress has been linked to increased production of pro-inflammatory cytokines [45]. Given the well-established connection between inflammation and central sensitization, stress-/anxiety-induced cytokine release may probably stand as one of the key contributors of increased DTR responses observed in fibromyalgia. Moreover, chronic anxiety can lead to alterations in the sympathetic nervous system [76]. Sympathetic activity can exert a direct impact on muscle spindles and facilitate the short-latency stretch reflex [77].

#### Hypomagnesaemia

Magnesium deficiency has been highlighted as a potential factor that plays role in the pathogenesis of fibromyalgia [78, 79]. Hypomagnesaemia is also associated with central nervous system hyperexcitability, which clinically manifests as an increase in DTR response. To such an extent that, DTR response can even be utilized for monitoring magnesium infusion and potential development of magnesium toxicity in pregnant women with eclampsia [80].

Magnesium suppresses the stimulated central nervous system by blocking N-Methyl-D-Aspartate receptors. Additionally, through its cerebral vasodilatory effects, magnesium prevents excitation caused by vasospasm in the central nervous system [80]. Therefore, low levels of magnesium lead to an increased excitation in the central nervous system, thus, hyperactive DTRs. In fibromyalgia, underlying hypomagnesaemia can contribute to increased DTR responses.

**Fig. 1:**
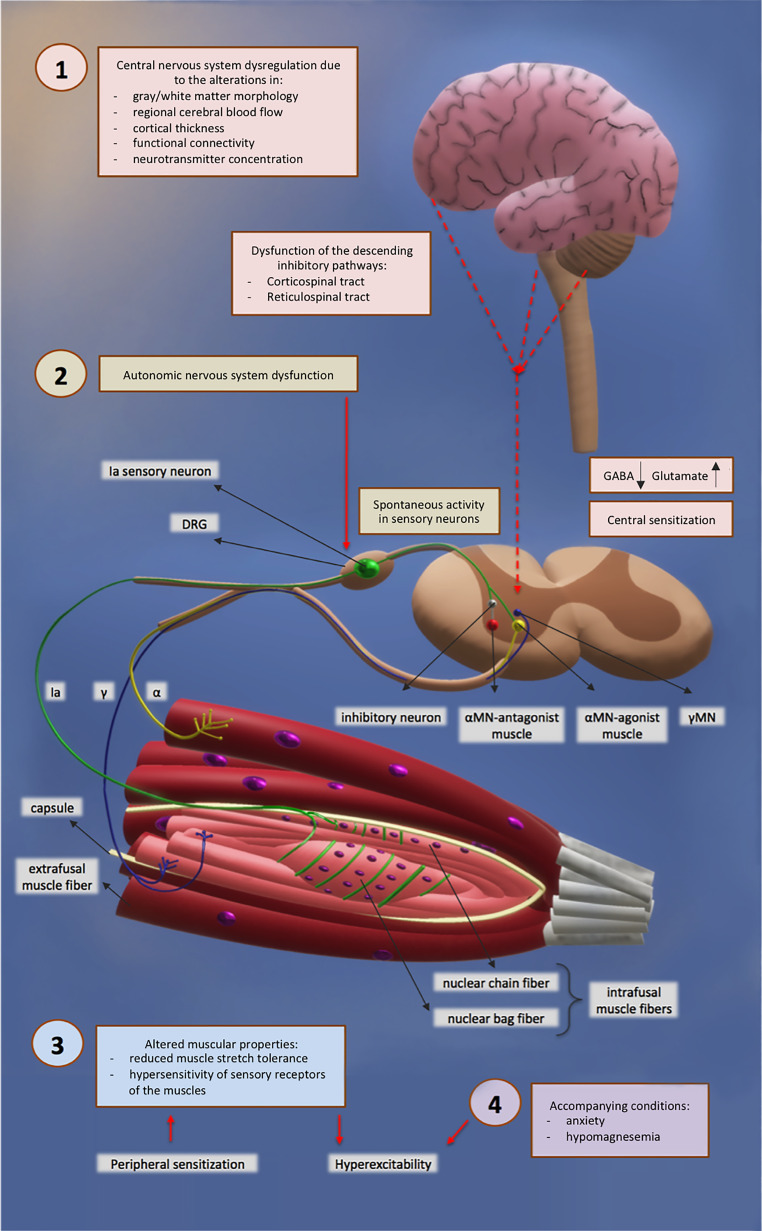
Potential mechanisms of increased deep tendon reflex responses in patients with fibromyalgia [27, 32, 51]. The figure was illustrated by the author, ICB. 1- Central nervous system dysregulation (hyperexcitability of spinal reflex arcs/central sensitization, disrupted inhibitory control), 2- Autonomic nervous system dysfunction, 3- Altered muscular properties, 4- Accompanying conditions (anxiety, hypomagnesemia). This illustration is not to scale and does not represent the real size, color, or proportions of cells and structures. MN: motor neuron, DRG: dorsal root ganglion

**Fig. 2:**
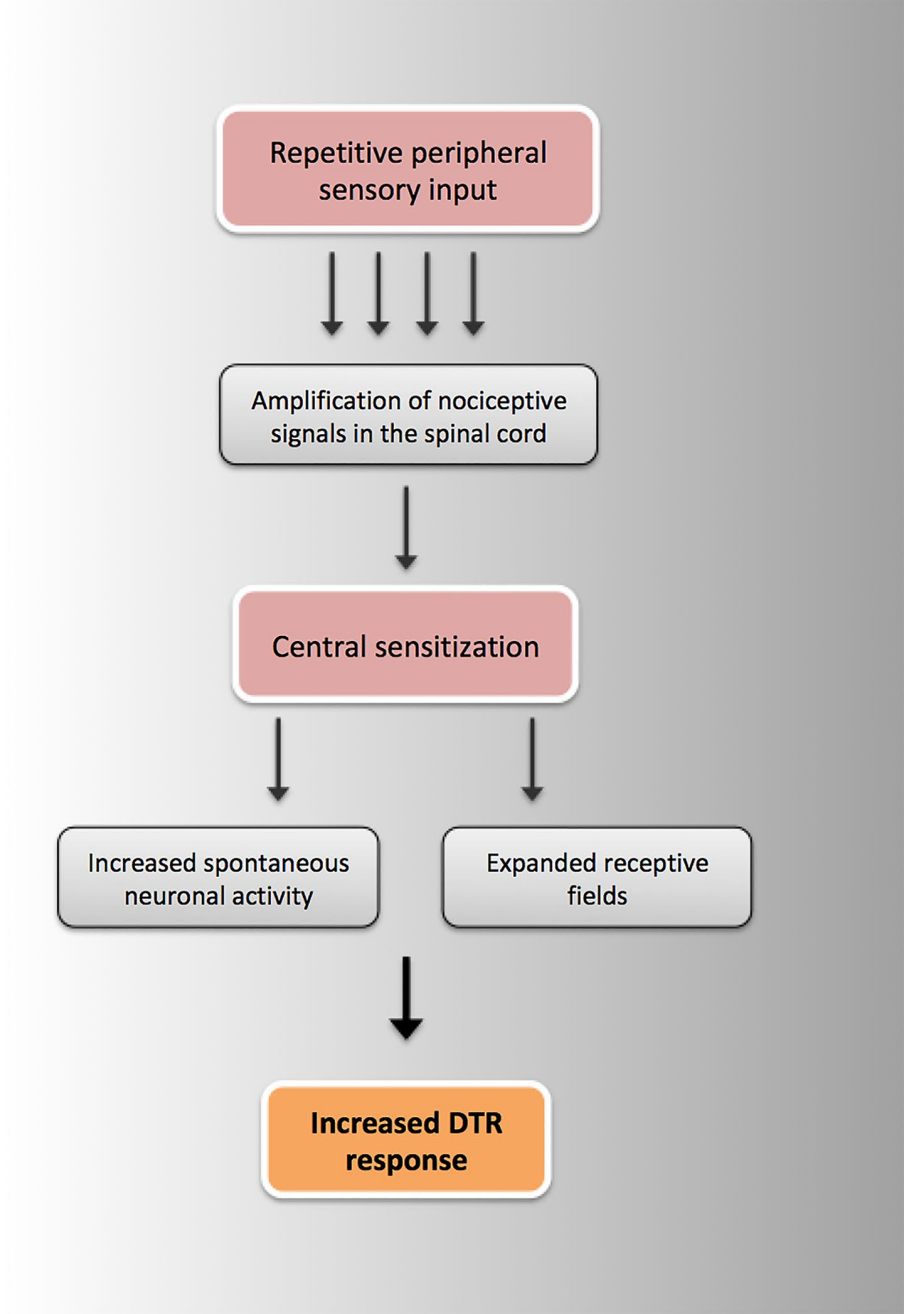
The potential pathway of hyperreflexia through central sensitization in patients with fibromyalgia [32, 34]. DTR: deep tendon reflex

The potential mechanisms underlying altered DTR responses in fibromyalgia were illustrated in Fig. 1. A detailed description of the potential role of central sensitization in the increased DTR responses was provided in Fig. 2.

## Exploring the integration of deep tendon reflex responses into current diagnostic criteria

Fibromyalgia is a multifaceted disorder and a challenging condition to diagnose, given its subjective symptomatology, which may overlap with other diseases. Current diagnostic criteria rely primarily on self-reported symptoms such as widespread pain, fatigue, sleep disturbances, and cognitive problems. The absence of an objective clinical test increases the risk of both underdiagnosis and overdiagnosis.

Given the recent evidence in fibromyalgia, we propose incorporating DTR response, as an objective neuromuscular marker, into the current diagnostic set. Our recent research demonstrated significantly higher reflex amplitudes, increased acceleration, and shorter electromechanical delay in patients with fibromyalgia. Normoactive or hypoactive DTR responses have been shown to have high sensitivity in ruling out fibromyalgia [1]. Implementing DTR responses into the diagnostic process of fibromyalgia offers a novel and objective approach that supplements the existing criteria. While current diagnostic tools rely on subjective patient reports, DTR responses provide measurable, quantifiable data that can help physicians differentiate fibromyalgia from other conditions with similar symptoms.

The addition of DTR examination to the diagnostic process of fibromyalgia has/may offer several advantages:

1) DTR assessment could be easily implemented in routine clinical evaluations.

2) DTR examination is a non-invasive and cost-effective tool.

3) The examination may serve as an objective and reproducible marker.

4) It may improve diagnostic accuracy by distinguishing fibromyalgia from other conditions that share similar symptom profiles.

5) The increase in the accuracy and reliability of fibromyalgia diagnosis would definitely improve patient outcomes.

While increased DTR responses are commonly observed in fibromyalgia, it is important to note that other conditions, such as neurological disorders (e.g., spinal cord lesions, stroke, multiple sclerosis) can also present with hyperreflexia. These conditions should be carefully considered when diagnosing fibromyalgia. Differential diagnosis is essential to avoid misdiagnosis. Therefore, physicians should perform a comprehensive evaluation of symptoms and medical history, as well as a detailed physical examination to ensure accurate diagnosis.

## Conclusions

Recent research has highlighted increased DTR responses in patients with fibromyalgia. Central sensitization, which is a key factor in fibromyalgia pathophysiology, may contribute to hyperexcitable spinal reflex arcs and reduced inhibitory control over DTR responses. Additionally, autonomic dysregulation may influence neuromuscular excitability.

The clinically and electrophysiologically proven increased DTR response in fibromyalgia may serve as a potential accelerator of diagnostic accuracy. Current diagnostic criteria for fibromyalgia, relying heavily on subjective patient reports, have some limitations. Incorporating DTR examination into the current diagnostic sets may not only add an objective, non-invasive, and reproducible marker to the criteria but also help differentiate fibromyalgia from other conditions. Further research should focus on how and to what extent this examination can be integrated into the existing diagnostic sets.

## Data Availability

Data sharing not applicable to this article as no datasets were generated or analyzed during the current study.

## Declarations

### Disclaimer

The authors state that no part of this manuscript, including the text and graphics, are copied or published elsewhere in whole or in part.
